# 2-Methyl-5,6-methyl­enedi­oxy-3-phenyl­sulfonyl-1-benzofuran

**DOI:** 10.1107/S160053680800980X

**Published:** 2008-04-16

**Authors:** Hong Dae Choi, Pil Ja Seo, Byeng Wha Son, Uk Lee

**Affiliations:** a Department of Chemistry, Dongeui University, San 24 Kaya-dong, Busanjin-gu, Busan 614-714, Republic of Korea; b Department of Chemistry, Pukyong National University, 599-1 Daeyeon 3-dong, Nam-gu, Busan 608-737, Republic of Korea

## Abstract

The title compound, C_16_H_12_O_5_S, was prepared by oxidation of 2-methyl-5,6-methyl­ene­di­oxy-3-phenyl­sulfanyl-1-benzo­furan with 3-chloro­peroxy­benzoic acid. The phenyl ring makes a dihedral angle of 83.64 (4)° with the mean plane of the 5,6-(methyl­ene­di­oxy)­benzo­furan fragment. The crystal structure is stabilized by C—H⋯π inter­actions between a benzene H atom of the 5,6-(methyl­ene­di­oxy)­benzo­furan unit and the phenyl ring of the phenyl­sulfonyl substituent. Additionally, the crystal structure exhibits inter- and intra­molecular C—H⋯O inter­actions.

## Related literature

For the crystal structures of similar 5,6-(methyl­ene­di­oxy)­benzo­furan compounds, see: Choi *et al.* (2007*a* ▶,*b* ▶).
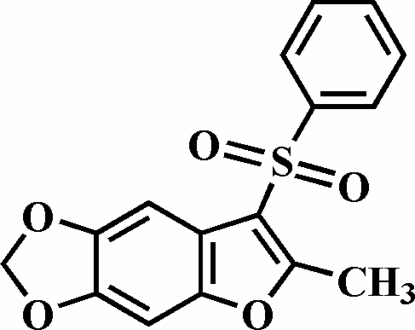


## Experimental

### 

#### Crystal data

C_16_H_12_O_5_S
*M* *_r_* = 316.32
Triclinic, 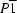

*a* = 7.4401 (3) Å
*b* = 8.8505 (4) Å
*c* = 11.2406 (5) Å
α = 89.801 (1)°
β = 72.565 (1)°
γ = 79.257 (1)°
*V* = 692.71 (5) Å^3^
*Z* = 2
Mo *K*α radiation
μ = 0.26 mm^−1^
*T* = 173 (2) K
0.40 × 0.20 × 0.20 mm

#### Data collection

Bruker SMART CCD diffractometer
Absorption correction: none
6000 measured reflections
2983 independent reflections
2746 reflections with *I* > 2σ(*I*)
*R*_int_ = 0.026

#### Refinement

*R*[*F* ^2^ > 2σ(*F* ^2^)] = 0.036
*wR*(*F* ^2^) = 0.096
*S* = 1.05
2983 reflections
200 parameters
H-atom parameters constrained
Δρ_max_ = 0.32 e Å^−3^
Δρ_min_ = −0.38 e Å^−3^

### 

Data collection: *SMART* (Bruker, 2001 ▶); cell refinement: *SAINT* (Bruker, 2001 ▶); data reduction: *SAINT*; program(s) used to solve structure: *SHELXS97* (Sheldrick, 2008 ▶); program(s) used to refine structure: *SHELXL97* (Sheldrick, 2008 ▶); molecular graphics: *ORTEP-3* (Farrugia, 1997 ▶) and *DIAMOND* (Brandenburg, 1998 ▶); software used to prepare material for publication: *SHELXL97*.

## Supplementary Material

Crystal structure: contains datablocks global, I. DOI: 10.1107/S160053680800980X/bh2167sup1.cif

Structure factors: contains datablocks I. DOI: 10.1107/S160053680800980X/bh2167Isup2.hkl

Additional supplementary materials:  crystallographic information; 3D view; checkCIF report

## Figures and Tables

**Table 1:** Hydrogen-bond geometry (Å, °) *Cg* is the centroid of the C9–C14 benzene ring.

*D*—H⋯*A*	*D*—H	H⋯*A*	*D*⋯*A*	*D*—H⋯*A*
C6—H6⋯*Cg*^i^	0.95	2.83	3.722 (3)	152
C3—H3⋯O4^ii^	0.95	2.53	3.379 (2)	150
C12—H12⋯O2^iii^	0.95	2.53	3.398 (2)	153
C16—H16*C*⋯O5	0.98	2.42	3.127 (3)	129

Symmetry codes: (i) 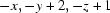; (ii) 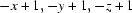; (iii) 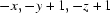.

**Fig. 1.**
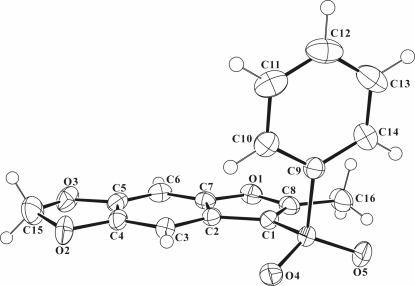
The molecular structure of the title compound, showing displacement ellipsoids drawn at the 50% probability level.

**Fig. 2.**
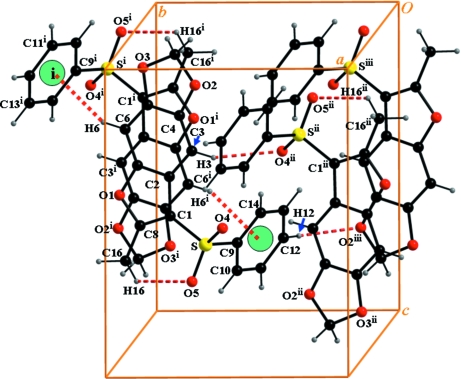
C—H···π and intra- and intermolecular C—H···O interactions (dotted lines) in the title compound. Cg denotes the ring centroids. [Symmetry code: (i) -x, -y + 2, -z + 1; (ii) -x + 1, -y + 1, -z + 1; iii) -x, -y + 1, -z + 1.]

## supplementary crystallographic information

### Comment

As part of our ongoing studies on the synthesis and structure of 5,6-(methylenedioxy)benzofuran derivatives, the crystal structures of 5,6-methylenedioxy-3-methylsulfinyl-2-phenylbenzofuran (Choi *et al.*, 2007*a*) and 2-methyl-5,6-methylenedioxy-3-methylsulfinyl-1-benzofuran (Choi *et al.*, 2007*b*) have been described to the literatures. Herein we report the molecular and crystal structure of the title compound, 2-methyl-5,6-methylenedioxy-3-phenylsulfonyl-1-benzofuran (Fig. 1).

The 5,6-(methylenedioxy)benzofuran unit is almost planar, with a mean deviation of 0.032 Å from the least-squares plane defined by the twelve constituent atoms. The crystal packing (Fig. 2) is stabilized by C—H···π interactions between a benzene H atom of 5,6-(methylenedioxy)benzofuran unit and the phenyl ring of the phenylsulfonyl substituent, with a C6—H6···*Cg*^i^ separation of 2.83 Å (Fig. 2 and Table 1; *Cg* is the centroid of the C9–C14 benzene ring, symmetry code as in Fig. 2). The molecular packing (Fig. 2) is further stabilized by intermolecular and intramolecular C—H···O interactions (Table 1 and Fig.2; symmetry codes as in Fig. 2).

### Experimental

3-Chloroperoxybenzoic acid (77%, 560 mg, 2.50 mmol) was added in small portions to a stirred solution of 2-methyl-5,6-methylenedioxy-3-phenyl-sulfonyl-1-benzofuran (341 mg, 1.20 mmol) in dichloromethane (30 ml) at room temperature. After being stirred for 4 h at room temperature, the mixture was washed with saturated sodium bicarbonate solution and the organic layer was separated, dried over magnesium sulfate, filtered and concentrated in vacuum. The residue was purified by column chromatography (hexane–ethyl acetate, 2:1 *v*/*v*) to afford the title compound as a colorless solid [yield 76%, m.p. 442–443 K; *R*_f_ = 0.54 (hexane–ethyl acetate, 2:1 *v*/*v*)]. Single crystals suitable for X-ray diffraction were prepared by slow evaporation of a solution of the title compound in acetone at room temperature.

### Refinement

All H atoms were positioned geometrically and refined using a riding model, with C—H = 0.95 Å for aromatic H atoms, 0.99 Å for methylene H atoms and 0.98 Å for methyl H atoms, respectively, and with *U*_iso_(H) = 1.2Ueq(C) for aromatic and methylene, *U*_iso_(H) = 1.5Ueq(C) for methyl H atoms.

### Crystal data

**Table:**

C_16_H_12_O_5_S	*Z* = 2
*M**_r_* = 316.32	*F*_000_ = 328
Triclinic, *P*1	*D*_x_ = 1.517 Mg m^−^^3^
Hall symbol: -P 1	Melting point = 442–443 K
*a* = 7.4401 (3) Å	Mo *K*α radiation λ = 0.71073 Å
*b* = 8.8505 (4) Å	Cell parameters from 4646 reflections
*c* = 11.2406 (5) Å	θ = 2.4–28.2º
α = 89.801 (1)º	µ = 0.26 mm^−^^1^
β = 72.565 (1)º	*T* = 173 (2) K
γ = 79.257 (1)º	Block, colourless
*V* = 692.71 (5) Å^3^	0.40 × 0.20 × 0.20 mm

### Data collection

**Table:**

Bruker SMART CCD diffractometer	2983 independent reflections
Radiation source: fine-focus sealed tube	2746 reflections with *I* > 2σ(*I*)
Monochromator: graphite	*R*_int_ = 0.026
Detector resolution: 10.0 pixels mm^-1^	θ_max_ = 27.0º
*T* = 173(2) K	θ_min_ = 2.9º
φ and ω scans	*h* = −9→9
Absorption correction: none	*k* = −11→11
6000 measured reflections	*l* = −14→13

### Refinement

**Table:**

Refinement on *F*^2^	Secondary atom site location: difference Fourier map
Least-squares matrix: full	Hydrogen site location: inferred from neighbouring sites
*R*[*F*^2^ > 2σ(*F*^2^)] = 0.036	H-atom parameters constrained
*wR*(*F*^2^) = 0.096	*w* = 1/[σ^2^(*F*_o_^2^) + (0.0449*P*)^2^ + 0.3658*P*] where *P* = (*F*_o_^2^ + 2*F*_c_^2^)/3
*S* = 1.05	(Δ/σ)_max_ < 0.001
2983 reflections	Δρ_max_ = 0.32 e Å^−^^3^
200 parameters	Δρ_min_ = −0.38 e Å^−^^3^
Primary atom site location: structure-invariant direct methods	Extinction correction: none

### Fractional atomic coordinates and isotropic or equivalent isotropic displacement parameters (Å^2^)

**Table:**

	*x*	*y*	*z*	*U*_iso_*/*U*_eq_	
S	0.36347 (5)	0.72181 (4)	0.70730 (3)	0.02587 (12)	
O1	0.16796 (16)	1.11741 (12)	0.58768 (11)	0.0317 (3)	
O2	0.46615 (19)	0.74239 (14)	0.16742 (10)	0.0364 (3)	
O3	0.3156 (2)	0.98837 (15)	0.14228 (11)	0.0399 (3)	
O4	0.52005 (16)	0.61904 (13)	0.62032 (10)	0.0313 (3)	
O5	0.38566 (18)	0.77361 (16)	0.82216 (11)	0.0388 (3)	
C1	0.2989 (2)	0.88120 (17)	0.62765 (14)	0.0250 (3)	
C2	0.3186 (2)	0.88013 (17)	0.49576 (14)	0.0236 (3)	
C3	0.4015 (2)	0.76992 (17)	0.39479 (13)	0.0249 (3)	
H3	0.4609	0.6676	0.4039	0.030*	
C4	0.3893 (2)	0.82284 (18)	0.28230 (14)	0.0266 (3)	
C5	0.3014 (2)	0.97239 (19)	0.26672 (15)	0.0290 (3)	
C6	0.2216 (2)	1.08254 (18)	0.36244 (16)	0.0311 (3)	
H6	0.1631	1.1847	0.3521	0.037*	
C7	0.2350 (2)	1.02901 (17)	0.47682 (15)	0.0263 (3)	
C8	0.2083 (2)	1.02469 (19)	0.67807 (15)	0.0299 (3)	
C9	0.1598 (2)	0.63445 (17)	0.74530 (14)	0.0259 (3)	
C10	0.1304 (2)	0.54383 (18)	0.65468 (16)	0.0311 (3)	
H10	0.2229	0.5230	0.5745	0.037*	
C11	−0.0363 (3)	0.4846 (2)	0.6836 (2)	0.0397 (4)	
H11	−0.0595	0.4236	0.6225	0.048*	
C12	−0.1687 (3)	0.5136 (2)	0.8009 (2)	0.0443 (5)	
H12	−0.2836	0.4739	0.8194	0.053*	
C13	−0.1355 (3)	0.6000 (2)	0.89174 (19)	0.0455 (5)	
H13	−0.2253	0.6162	0.9731	0.055*	
C14	0.0288 (3)	0.6630 (2)	0.86401 (16)	0.0366 (4)	
H14	0.0512	0.7245	0.9252	0.044*	
C15	0.4011 (3)	0.8388 (2)	0.08173 (17)	0.0467 (5)	
H15A	0.3056	0.7952	0.0546	0.056*	
H15B	0.5103	0.8463	0.0071	0.056*	
C16	0.1447 (3)	1.0983 (2)	0.80603 (17)	0.0421 (4)	
H16A	0.0064	1.1036	0.8430	0.063*	
H16B	0.1720	1.2027	0.8021	0.063*	
H16C	0.2137	1.0374	0.8574	0.063*	

### Atomic displacement parameters (Å^2^)

**Table:**

	*U*^11^	*U*^22^	*U*^33^	*U*^12^	*U*^13^	*U*^23^
S	0.0256 (2)	0.0340 (2)	0.01994 (19)	−0.00685 (15)	−0.00916 (14)	0.00132 (14)
O1	0.0297 (6)	0.0249 (5)	0.0371 (6)	−0.0040 (4)	−0.0060 (5)	−0.0037 (5)
O2	0.0539 (8)	0.0377 (6)	0.0213 (5)	−0.0141 (6)	−0.0137 (5)	0.0014 (5)
O3	0.0539 (8)	0.0439 (7)	0.0329 (6)	−0.0191 (6)	−0.0238 (6)	0.0157 (5)
O4	0.0265 (6)	0.0366 (6)	0.0286 (6)	−0.0008 (5)	−0.0082 (4)	0.0029 (5)
O5	0.0428 (7)	0.0556 (8)	0.0249 (6)	−0.0163 (6)	−0.0164 (5)	0.0010 (5)
C1	0.0237 (7)	0.0285 (7)	0.0236 (7)	−0.0070 (6)	−0.0071 (5)	−0.0009 (6)
C2	0.0223 (7)	0.0257 (7)	0.0243 (7)	−0.0070 (5)	−0.0080 (5)	0.0024 (5)
C3	0.0284 (7)	0.0238 (7)	0.0236 (7)	−0.0054 (6)	−0.0091 (6)	0.0014 (6)
C4	0.0299 (7)	0.0295 (8)	0.0236 (7)	−0.0124 (6)	−0.0090 (6)	0.0024 (6)
C5	0.0296 (8)	0.0355 (8)	0.0291 (8)	−0.0148 (6)	−0.0145 (6)	0.0115 (6)
C6	0.0284 (8)	0.0262 (7)	0.0418 (9)	−0.0072 (6)	−0.0142 (7)	0.0107 (7)
C7	0.0226 (7)	0.0244 (7)	0.0319 (8)	−0.0065 (5)	−0.0069 (6)	−0.0002 (6)
C8	0.0252 (7)	0.0329 (8)	0.0304 (8)	−0.0093 (6)	−0.0044 (6)	−0.0045 (6)
C9	0.0254 (7)	0.0273 (7)	0.0257 (7)	−0.0053 (6)	−0.0089 (6)	0.0048 (6)
C10	0.0328 (8)	0.0276 (8)	0.0336 (8)	−0.0028 (6)	−0.0131 (7)	0.0014 (6)
C11	0.0406 (9)	0.0276 (8)	0.0580 (11)	−0.0083 (7)	−0.0244 (9)	0.0041 (8)
C12	0.0325 (9)	0.0333 (9)	0.0694 (13)	−0.0113 (7)	−0.0160 (9)	0.0172 (9)
C13	0.0332 (9)	0.0470 (11)	0.0467 (11)	−0.0073 (8)	0.0017 (8)	0.0132 (8)
C14	0.0366 (9)	0.0406 (9)	0.0290 (8)	−0.0078 (7)	−0.0045 (7)	0.0023 (7)
C15	0.0520 (11)	0.0598 (12)	0.0258 (9)	−0.0075 (9)	−0.0105 (8)	0.0105 (8)
C16	0.0381 (9)	0.0464 (10)	0.0364 (9)	−0.0107 (8)	−0.0017 (7)	−0.0164 (8)

### Geometric parameters (Å, °)

**Table:**

S—O5	1.4369 (12)	C6—H6	0.9500
S—O4	1.4384 (12)	C8—C16	1.484 (2)
S—C1	1.7374 (16)	C9—C14	1.388 (2)
S—C9	1.7674 (15)	C9—C10	1.390 (2)
O1—C8	1.371 (2)	C10—C11	1.385 (2)
O1—C7	1.3794 (18)	C10—H10	0.9500
O2—C4	1.3833 (18)	C11—C12	1.380 (3)
O2—C15	1.418 (2)	C11—H11	0.9500
O3—C5	1.3797 (19)	C12—C13	1.384 (3)
O3—C15	1.433 (2)	C12—H12	0.9500
C1—C8	1.358 (2)	C13—C14	1.388 (3)
C1—C2	1.445 (2)	C13—H13	0.9500
C2—C7	1.393 (2)	C14—H14	0.9500
C2—C3	1.409 (2)	C15—H15A	0.9900
C3—C4	1.369 (2)	C15—H15B	0.9900
C3—H3	0.9500	C16—H16A	0.9800
C4—C5	1.398 (2)	C16—H16B	0.9800
C5—C6	1.367 (2)	C16—H16C	0.9800
C6—C7	1.394 (2)		
			
O5—S—O4	119.53 (7)	O1—C8—C16	115.53 (15)
O5—S—C1	108.96 (8)	C14—C9—C10	121.50 (15)
O4—S—C1	107.55 (7)	C14—C9—S	118.94 (13)
O5—S—C9	107.76 (7)	C10—C9—S	119.51 (12)
O4—S—C9	108.26 (7)	C11—C10—C9	118.71 (16)
C1—S—C9	103.65 (7)	C11—C10—H10	120.6
C8—O1—C7	107.02 (12)	C9—C10—H10	120.6
C4—O2—C15	105.85 (13)	C12—C11—C10	120.33 (17)
C5—O3—C15	105.87 (13)	C12—C11—H11	119.8
C8—C1—C2	107.71 (14)	C10—C11—H11	119.8
C8—C1—S	126.84 (12)	C11—C12—C13	120.56 (17)
C2—C1—S	125.26 (11)	C11—C12—H12	119.7
C7—C2—C3	120.48 (14)	C13—C12—H12	119.7
C7—C2—C1	104.66 (13)	C12—C13—C14	120.04 (18)
C3—C2—C1	134.85 (14)	C12—C13—H13	120.0
C4—C3—C2	114.24 (14)	C14—C13—H13	120.0
C4—C3—H3	122.9	C9—C14—C13	118.81 (17)
C2—C3—H3	122.9	C9—C14—H14	120.6
C3—C4—O2	126.58 (14)	C13—C14—H14	120.6
C3—C4—C5	123.90 (14)	O2—C15—O3	108.47 (14)
O2—C4—C5	109.48 (13)	O2—C15—H15A	110.0
C6—C5—O3	127.47 (15)	O3—C15—H15A	110.0
C6—C5—C4	123.31 (14)	O2—C15—H15B	110.0
O3—C5—C4	109.19 (14)	O3—C15—H15B	110.0
C5—C6—C7	112.80 (14)	H15A—C15—H15B	108.4
C5—C6—H6	123.6	C8—C16—H16A	109.5
C7—C6—H6	123.6	C8—C16—H16B	109.5
O1—C7—C2	110.33 (13)	H16A—C16—H16B	109.5
O1—C7—C6	124.42 (14)	C8—C16—H16C	109.5
C2—C7—C6	125.25 (15)	H16A—C16—H16C	109.5
C1—C8—O1	110.29 (14)	H16B—C16—H16C	109.5
C1—C8—C16	134.18 (17)		
			
O5—S—C1—C8	−25.07 (16)	C1—C2—C7—O1	0.06 (16)
O4—S—C1—C8	−156.01 (14)	C3—C2—C7—C6	1.1 (2)
C9—S—C1—C8	89.47 (15)	C1—C2—C7—C6	179.64 (14)
O5—S—C1—C2	160.50 (12)	C5—C6—C7—O1	179.15 (13)
O4—S—C1—C2	29.55 (15)	C5—C6—C7—C2	−0.4 (2)
C9—S—C1—C2	−84.96 (14)	C2—C1—C8—O1	−0.16 (17)
C8—C1—C2—C7	0.06 (16)	S—C1—C8—O1	−175.39 (11)
S—C1—C2—C7	175.39 (11)	C2—C1—C8—C16	179.14 (17)
C8—C1—C2—C3	178.25 (16)	S—C1—C8—C16	3.9 (3)
S—C1—C2—C3	−6.4 (3)	C7—O1—C8—C1	0.19 (17)
C7—C2—C3—C4	−0.6 (2)	C7—O1—C8—C16	−179.25 (13)
C1—C2—C3—C4	−178.60 (15)	O5—S—C9—C14	17.03 (15)
C2—C3—C4—O2	176.68 (14)	O4—S—C9—C14	147.62 (13)
C2—C3—C4—C5	−0.5 (2)	C1—S—C9—C14	−98.37 (14)
C15—O2—C4—C3	174.65 (16)	O5—S—C9—C10	−165.42 (12)
C15—O2—C4—C5	−7.83 (18)	O4—S—C9—C10	−34.83 (14)
C15—O3—C5—C6	−177.05 (17)	C1—S—C9—C10	79.18 (13)
C15—O3—C5—C4	4.93 (18)	C14—C9—C10—C11	1.7 (2)
C3—C4—C5—C6	1.3 (2)	S—C9—C10—C11	−175.83 (12)
O2—C4—C5—C6	−176.29 (14)	C9—C10—C11—C12	−0.9 (2)
C3—C4—C5—O3	179.42 (14)	C10—C11—C12—C13	−1.1 (3)
O2—C4—C5—O3	1.82 (17)	C11—C12—C13—C14	2.3 (3)
O3—C5—C6—C7	−178.56 (14)	C10—C9—C14—C13	−0.5 (3)
C4—C5—C6—C7	−0.8 (2)	S—C9—C14—C13	177.04 (14)
C8—O1—C7—C2	−0.15 (16)	C12—C13—C14—C9	−1.5 (3)
C8—O1—C7—C6	−179.74 (14)	C4—O2—C15—O3	10.86 (19)
C3—C2—C7—O1	−178.45 (12)	C5—O3—C15—O2	−9.81 (19)

### Hydrogen-bond geometry (Å, °)

**Table:**

*D*—H···*A*	*D*—H	H···*A*	*D*···*A*	*D*—H···*A*
C6—H6···Cg^i^	0.95	2.83	3.722 (3)	152
C3—H3···O4^ii^	0.95	2.53	3.379 (2)	150
C12—H12···O2^iii^	0.95	2.53	3.398 (2)	153
C16—H16C···O5	0.98	2.42	3.127 (3)	129

Symmetry codes: (i) −*x*, −*y*+2, −*z*+1; (ii) −*x*+1, −*y*+1, −*z*+1; (iii) −*x*, −*y*+1, −*z*+1.
